# Study protocol: the effects of air pollution exposure and chronic respiratory disease on pneumonia risk in urban Malawian adults - the Acute Infection of the Respiratory Tract Study (The AIR Study)

**DOI:** 10.1186/s12890-015-0090-3

**Published:** 2015-08-20

**Authors:** Hannah Jary, Jane Mallewa, Mulinda Nyirenda, Brian Faragher, Robert Heyderman, Ingrid Peterson, Stephen Gordon, Kevin Mortimer

**Affiliations:** Malawi-Liverpool-Wellcome Trust Clinical Research Programme, University of Malawi College of Medicine, Blantyre, Malawi; College of Medicine, University of Malawi, Blantyre, Malawi; Queen Elizabeth Central Hospital, Ministry of Health, Blantyre, Malawi; Liverpool School of Tropical Medicine, Liverpool, UK

**Keywords:** Pneumonia, Air pollution, Chronic respiratory disease, Africa, Particulate matter, Carbon monoxide, Spirometry

## Abstract

**Background:**

Pneumonia is the 2nd leading cause of years of life lost worldwide and is a common cause of adult admissions to hospital in sub-Saharan Africa. Risk factors for adult pneumonia are well characterised in developed countries, but are less well described in sub-Saharan Africa where HIV is a major contributing factor. Exposure to indoor and outdoor air pollution is high, and tobacco smoking prevalence is increasing in sub-Saharan Africa, yet the contribution of these factors to the burden of chronic respiratory diseases in sub-Saharan Africa remains poorly understood. Furthermore, the extent to which the presence of chronic respiratory diseases and exposure to air pollution contribute to the burden of pneumonia is not known.

**Design:**

The Acute Infection of the Respiratory Tract Study (The AIR Study) is a case–control study to identify preventable risk factors for adult pneumonia in the city of Blantyre, Malawi. Cases will be adults admitted with pneumonia, recruited from Queen Elizabeth Central Hospital, the largest teaching hospital in Malawi. Controls will be adults without pneumonia, recruited from the community. The AIR Study will recruit subjects and analyse data within strata defined by positive and negative HIV infection status. All participants will undergo thorough assessment for a range of potential preventable risk factors, with an emphasis on exposure to air pollution and the presence of chronic respiratory diseases. This will include collection of questionnaire data, clinical samples (blood, urine, sputum and breath samples), lung function data and air pollution monitoring in their home. Multivariate analysis will be used to identify the important risk factors contributing to the pneumonia burden in this setting. Identification of preventable risk factors will justify research into the effectiveness of targeted interventions to address this burden in the future.

**Discussion:**

The AIR Study is the first study of radiologically confirmed pneumonia in which air pollution exposure measurements have been undertaken in this setting, and will contribute important new information about exposure to air pollution in urban SSA. Through identification of preventable risk factors, the AIR Study aims to facilitate future research and implementation of targeted interventions to reduce the high burden of pneumonia in SSA.

## Background

Pneumonia is a leading cause of premature death worldwide [1], and a common cause of adult admission to hospital in sub-Saharan Africa (SSA) in both the pre- and post-HIV eras [2, 3]. Adult pneumonia mortality in SSA varies between 6 and 14.5 % [4–6], with over 50 % of deaths in under 35 year olds [5].

Risk factors for developing pneumonia in affluent countries are well described but are less well understood in countries like Malawi where HIV is known to be a major contributing factor [7–9]. As HIV management improves and living with HIV becomes life with a chronic disease, risk factors for pneumonia other than HIV will become increasingly important. Poverty and crowded living conditions have previously been identified as risk factors for adult pneumonia in SSA [10, 11]. Many other preventable exposures, including chronic respiratory disease (CRD), air pollution exposure, tobacco smoke, malnutrition, excess alcohol and animal ownership [12–22], may play a role but their relative importance in SSA is uncertain.

With an ageing global population, there is a well recognised emerging global epidemic of non-communicable diseases, with the highest burden expected in SSA [23]. The burden of CRD - referring to any persistent disease of the lower respiratory tract, including chronic obstructive pulmonary disease (COPD), chronic bronchitis, bronchiectasis, and asthma - is unknown in SSA [24] but expected to be high, due to multiple interacting insults throughout an individual’s lifetime, including maternal and childhood malnutrition, recurrent respiratory tract infections, HIV, tuberculosis (TB), air pollution exposures and tobacco smoke.

According to the Global Burden of Disease Study, tobacco smoke, indoor air pollution and outdoor air pollution are the 2^nd^, 4^th^ and 9^th^ leading global risk factors for disability-adjusted life years (DALYs) respectively [24]. 95 % of people in Malawi use unprocessed biomass fuels (wood, charcoal, animal dung, crop residues) as their main source of energy for essential household activities [26], resulting in pollutant levels inside homes much greater than the World Health Organisation (WHO) considers safe [27]. Household air pollution (HAP) is a recognised risk factor for childhood pneumonia [15], but the evidence for adults is less conclusive: the Global Burden of Disease study 2010 did not include estimates for this effect in their analysis due to the lack of available evidence [25, 28]. Although tobacco smoking is a well-recognised risk factor for pneumonia in developed countries [17], very few studies in SSA have evaluated this association.

Pneumonia causes a major health burden in sub-Saharan African adults, yet the risk factors for this are poorly understood. This report outlines the protocol for a study which aims to identify preventable risk factors which will provide justification for targeted interventions, to reduce the burden of disease seen. Robust data regarding the association between inhalable pollutants and adult pneumonia will inform health policy and ensure appropriate allocation of resources towards intervention strategies to reduce exposure: implementation of air quality guidelines, indoor and outdoor pollution reduction and smoking prevention strategies. If an association is demonstrated, strategies to prevent CRD could not only help to reduce CRD, but may also impact on the pneumonia burden.

**The primary objectives of the study are:**

- To determine the odds ratio of hospitalised adult pneumonia by exposure to air pollution.
- To determine the odds ratio of hospitalised adult pneumonia by the presence of CRD.

## Methods/Design

### Study design and setting

The Acute Infection of the Respiratory tract Study (the AIR Study) is a case–control study set in Blantyre, Malawi’s second largest city, where the urban population has an estimated HIV prevalence of 17.4 %. Queen Elizabeth Central Hospital (QECH), from where cases will be recruited, is a large teaching hospital providing healthcare to greater Blantyre (population approximately 1 million). Over 600 adult patients with pneumonia are admitted to QECH per year and HIV prevalence in these cases is approximately 70 %. Cases and controls will be frequency matched by age and gender, and the study will recruit subjects and analyse data within strata defined by positive and negative HIV infection status. All participants will undergo assessment for CRD and detailed air pollution exposure monitoring (indoor and outdoor) at their homes.

### Case definition

Cases will be recruited from QECH and defined by the presence of the outcome of interest (pneumonia requiring hospitalisation), using the inclusion and exclusion criteria shown in Table 1.

**Table 1:** Inclusion and exclusion criteria for cases and controls

Criteria for cases		Criteria for controls	
Inclusion	Exclusion	Inclusion	Exclusion
Age 18 or over	Pre-admission diagnosis of terminal illness (e.g. metastatic malignancy, terminal AIDS)	Age 18 or over	Pre-existing diagnosis of terminal illness (e.g. metastatic malignancy, terminal AIDS)
Lives in Blantyre city	Current anti-TB treatment or evidence of current TB infection	Lives in Blantyre city	Current anti-TB treatment or evidence of current TB infection
Reported cough or chest pain or breathlessness or haemoptysis	Prior hospitalisation within the last 4 weeks		Hospitalisation for a pneumonia-like illness in the past 4 months or current pneumonia-like illness
Reported fever or recorded fever (≥38 °C)	Prior participation in the study		Prior participation in the study
Crepitations or pleural rub or bronchial breathing or clinical features of pleural effusion	Lives in a residential institution (eg. prison)		Lives in a residential institution (eg. prison)
Radiological changes judged to be new and consistent with pneumonia, without another obvious cause	Death prior to follow up assessment		Death prior to follow up assessment
Requires hospitalisation	Alternative diagnosis explaining their presentation		Utilises private health care facilities if has illness requiring hospitalisation
	Symptoms for 14 days or more		

Due to possible delays in obtaining chest x-rays for some patients, individuals will be recruited if they meet the other eligibility criteria prior to x-ray, and then will be subsequently excluded if they do not have radiological changes consistent with pneumonia.

Recruited individuals with pneumonia will be subsequently excluded as ‘case’ if they have died or commenced anti-tuberculous treatment prior to completion of their follow up assessment. These individuals will be excluded from the final analysis but data will be retained for sub-group analysis.

### Control definition

Controls will be recruited from the community and defined by the absence of the outcome of interest (pneumonia requiring hospitalisation), using the inclusion and exclusion criteria shown in Table 1.

### Stratification and matching

Recruitment will be stratified by HIV status, which will enable data to be analysed as two separate cases-control studies: a HIV-positive case–control sub-study, and a HIV-negative case–control sub-study. Within these two separate sub-studies, controls will be frequency matched to cases by HIV age strata and gender. This will be done by randomly selecting control within strata of matching factors at a frequency determined by recent pneumonia studies at QECH. Cases and controls will be recruited concurrently in order to frequency match by season. Matching factors will be controlled for in data analysis.

### Study group recruitment

#### Case identification and recruitment

Adults admitted to QECH will be screened for symptoms suggestive of pneumonia by study staff. Identified patients and their case notes will be reviewed by the study clinical officer or principal investigator for eligibility for the study. Written informed consent from the patient or proxy consent from their accompanying guardian will be obtained in their first language.

#### Control identification and recruitment

Within Blantyre City, community controls will be randomly selected by residential census enumeration area (EA), where EAs are randomly chosen weighted by population density. (EA boundary and population data were obtained from National Office of Statistics, Malawi). Random Global Positioning System (GPS) starting points and vectors within the chosen neighbourhoods will be generated and overlaid onto high resolution satellite maps of Blantyre EAs using Google Earth pro software. To identify controls, field workers will start from the randomly generated GPS point, visiting the first dwelling intersected by the randomly generated vector. All potential control participants in that dwelling will be identified, screened for eligibility (including age strata and gender) and asked for written consent to perform a HIV test using a rapid diagnostic test. Pre- and post-test counselling will be provided. Individuals eligible for the study, will be asked to provide written informed consent to join the study. Illiterate participants will be consented in the presence of a literate independent witness, who will countersign the participant’s thumbprint. Where multiple individuals within the same household are eligible, an individual will be randomly chosen for recruitment. A maximum of one individual will be recruited per household.

If no one is home, dwellings will be visited up to three times. Where an individual meeting eligibility criteria has been identified but is not home, up to three attempts to locate that individual will be made.

The next dwelling intersected by the vector will then be visited, and the process repeated. If the study team reach the EA border, recruitment will stop and a new randomly generated starting point within the same residential neighbourhood will be used. Two controls will be recruited from each EA, unless the EA has been randomly chosen more than once.

The primary strategy for control recruitment, outlined above, will also be supplemented by recruitment of HIV positive individuals from Anti-retroviral Clinics at community Health Centres within Blantyre. It is anticipated that approximately one third of HIV positive controls will be recruited using this method (approximately 50 individuals). This supplementary method will be used to ensure that adequate numbers of HIV positive controls will be recruited to the study, as the above door-to-door method is resource intensive and identification of HIV positive controls is challenging. The six health centres from across Blantyre will be visited in rotation by study staff, and new and existing patients at these clinics will be invited to participate.

### Study procedures

#### In-hospital assessment of cases

Initial assessment of cases will include:

- Medical history using questionnaires and case notes
- Clinical examination
  - ○ blood pressure, pulse, temperature, respiratory rate, oxygen saturations
  - ○ height and weight (to calculate Body Mass Index (BMI))
  - ○ respiratory, cardiovascular and abdominal examination
  - ○ assessment of cognitive function
- Chest x-ray
- Blood culture
- Malaria rapid antigen test
- HIV testing and CD4 count
- Sputum specimen for tuberculous diagnostics - smear fluorescent microscopy for acid-alcohol fast bacilli, culture and GeneXpert®. If no spontaneous sputum sample obtained, sputum induction using nebulised hypertonic saline will be used when clinically indicated.
- Diagnostic thoracocentesis (if pleural effusion is present on chest x-ray and is clinically appreciable) for tuberculous diagnostics - smear fluorescent microscopy for acid-alcohol fast bacilli and culture.
- BinaxNOW® *Streptococcus pneumoniae* urinary antigen test

#### Exposure assessment in cases and controls

Exposure assessments will be made in the participant’s home. Cases will undergo this assessment 2–4 months following their admission for pneumonia (to allow time to recover from their acute illness and return to usual activities), and controls will be followed up within 1–2 weeks of recruitment. Exposure assessments will include:

- Questionnaires:
  - ○ BOLD Questionnaires (Burden of Obstructive Lung Disease Study, www.boldstudy.org) – sections of the BOLD questionnaires will be used to assess risk factors including socioeconomic factors, chronic respiratory symptoms, comorbidities and smoking history.
  - ○ An additional questionnaire will be used to assess other potential risk factors (including indoor air pollution exposure, occupational smoke exposures, contact with children / people with illnesses, household conditions and alcohol)
- Clinical measurements:
  - ○ blood pressure, pulse, temperature, respiratory rate, oxygen saturations
  - ○ spirometry – pre- and post-bronchodilator Forced Expiratory Volume in 1 s (FEV_1_) and Forced Vital Capacity (FVC) will be measured using an NDD EasyOne Spirometer according to ATS/ERS guidelines [29]
    - ▪ if spirometry is abnormal in cases then this will be repeated following a 3–4 months period to assess whether the abnormality is a chronic or due to incomplete recovery from the pneumonia episode.
  - ○ sputum eosinophil counts will be assessed (in a subsequent additional hospital visit) in a subset of participants, using sputum induction with nebulised hypertonic saline.
- Air pollution exposure assessment:
  - ○ exhaled Carbon Monoxide (CO) measurement, using a MicroCO monitor.
  - ○ composite air pollution exposure monitoring:
    - ▪ 48 h ambulatory for Particulate Matter <2.5 μm in diameter (PM_2.5)_ and CO monitoring: participants will be asked to wear an Aprovecho Indoor Air Pollution Meter in a backpack for continuous monitoring.
    - ▪ 48 h household PM_2.5_ and CO monitoring: UCB-PATS (University of California, Berkeley - Particle and Temperature Sensor) and Lascar EL-USB-CO Data Logger monitors will be placed in the participants kitchen or cooking areas.

During this follow up visit, controls will undergo the following additional assessments (which have been completed during the in-patient admission for cases):

- height and weight
- HIV test and CD_4_ count
- Sputum induction for tuberculous diagnostics if chronic cough present suggestive of possible TB.

### Exposure variables

#### Primary chronic respiratory disease exposure of interest

- A composite assessment of evidence of CRD, defined by:
  - ○ history of chronic respiratory symptoms in keeping with COPD, bronchitis bronchiectasis or asthma, as evidenced by one or both of the following:
    - ▪ answering in the affirmative to BOLD questions regarding cough, phlegm, wheezing or breathlessness.
    - ▪ reporting a current diagnosis and treatment of emphysema, asthma, asthmatic bronchitis, allergic bronchitis, chronic bronchitis or COPD (as per the BOLD questionnaire).

#### Primary air pollution exposure of interest

- A composite assessment of indoor and outdoor air pollution exposure:
  - ○ Area under the curve of personal PM_2.5_ exposure over a 48 h period

#### Secondary exposures and covariates of interest

- History of previous respiratory disease
- Area under the curve of personal CO exposure over a 48 h period
- Mean/median and peak personal CO exposure over a 48 h
- Mean/median and peak personal PM_2.5_ exposure over a 48 h
- Area under the curve of household PM_2.5_ exposure over a 48 h period
- Area under the curve of household CO exposure over a 48 h period
- Mean/median and peak household CO exposure over a 48 h
- Mean/median and peak household PM_2.5_ exposure over a 48 h
- Self-reported air pollution exposures
  - ○ Including HAP, occupational and tobacco smoke exposure (personal and passive)
- HIV status, CD4 count, antiretroviral therapy use
- Spirometric evidence of obstruction or restriction
- Exhaled Carbon Monoxide
- Evidence of airway inflammation
- Malnutrition (BMI)
- Alcohol use
- Vaccination history
- Poverty indicators
- Crowding
- Contact with children
- Contact with people with illnesses
- Animal ownership
- Marital status

### Data collection, management and storage

Collection of data and storage will comply with Good Clinical Practice guidelines as defined by the International Conference on Harmonisation guidelines. All questionnaire and examination data will initially be collected on hard copy Case Report Forms (CRFs) which will only be identifiable by a barcode containing the participant’s unique study number. Intelligent character recognition scanning software (Teleform®) will be used to convert data into an electronic format. Laboratory samples will be labelled with barcode and study number, and results will be electronically transferred to the study database. Air pollution monitoring data will be downloaded directly from the monitoring equipment for analysis with the relevant software. Chest x-ray films will be captured by digital photograph, and will be stored anonymously for radiological analysis.

CRFs will be stored in locked filling cabinets within MLW, only accessible to the study team and the MLW data management group. All electronic data will be exported to a database on the secure MLW server. Data files will be exported to Stata 13.1 (Statacorp, USA) for analysis. Access to the study database will be password-protected and restricted to the principal investigator, study investigators, and study data manager. Analysis of anonymised electronic data will be performed by the principal investigator, with support from co-investigators and the study statistician.

### Quality control

To ensure delivery of a high quality study, the following measures will be taken:

- Standardised methodology will be employed for participant recruitment and data collection, and Standard Operating Procedures will be followed for all necessary procedures.
- All study workers will be provided with study-specific training prior to study commencement and performance will be monitored throughout the study.
- Spirometry will be performed by individuals who are certified to perform spirometry to international standards.
- All study workers will complete GCP training prior to study commencement.
- All equipment will be regularly calibrated in line with manufacturer’s recommendations.
- Spirometry tracing and radiology will be reviewed independently by a 2^nd^ researcher with appropriate expertise.

### Statistical considerations

#### Sample size

Assumptions:

- Percentage of controls with exposure of interest (eg. CRD) 15–50 %
- α = 0.05
- β = 0.02

The target sample sizes for exposure assessment in the two sub-studies are:

- HIV-positive sub-study (case:control ratio 1:1) to detect an odds ratio of 1.9–2.2:
  - ○ Cases 160
  - ○ Controls 160
- HIV-negative sub-study (case:control ratio 1:1.5) to detect an odds ratio of 2.7–3.0:
  - ○ Cases 60
  - ○ Controls 90

Up to 50 % of recruited cases are expected to be excluded following recruitment but prior to follow up (due to lack of radiological pneumonia, deaths and commencing TB treatment). To account for this expected loss to follow up, the total recruitment targets to achieve these follow up targets are:

- ○ cases 524
- ○ controls 278

#### Analysis plan

HIV is expected to have a large impact on the outcome, and potential interactions between HIV and other risk factors for pneumonia may exist. Within the resource constraints of this study, it is unlikely that this study will be adequately powered to explore these interactions. The study will therefore be analysed as two separate case–control studies; a HIV-positive study and a HIV-negative study.

Univariate comparisons between groups will be made for explanatory variables using two-sample *t*-tests or Mann–Whitney tests for continuous data or Pearson’s Chi-square / Fisher’s exact testing for categorical data (*p* ≤0.05 will be considered statistically significant). Correlations between continuous explanatory variables will be tested for using Pearson’s or Spearman’s rank correlation tests. Explanatory variables that are associated with pneumonia with a p value of less than 0.2 will be eligible for inclusion in a multivariate regression model. An unconditional logistic regression model will be developed using a stepwise forward selection approach (criteria for entry *p* < 0.05 and removal *p* < 0.1), to identify risk factors for pneumonia incorporating adjustment for potential confounders as well as matching factors (matching factors will be treated as nuisance parameters and their coefficients will not be reported). Interaction terms of interest will be tested prior to finalising the model. The assumptions of the model will be tested by examination of the residuals, and the overall fit of the model will be ascertained using the Hosmer and Lemeshow goodness-of-fit model.

Structural equation modelling will be carried out to enable assessment of causal pathways and confounding. This methodology enables a quantitative estimation of relationships between the predictor variables, as well as between the predictor variables and the outcome variable.

Sensitivity analyses will be performed, to assess for the effects of exclusion of individuals who did not survive and inclusion of cases with multiple episodes of hospitalised pneumonia.

A comparison of characteristics between the controls recruited by the primary strategy (household recruitment) and the supplementary strategy (clinic recruitment) will be made. If significant differences are found, a sensitivity analysis will be performed to assess the effects of this on the findings.

Secondary analyses will explore risk factors for pneumonia severity within cases.

### Ethical considerations

This study will be carried out to include the protection of human subjects according to the 2008 Declaration of Helsinki and in accordance with Good Clinical Practice guidelines. Informed consent will be obtained from all study participants (or their guardian) in their first language, either written or witnessed verbal consent if the participant (or guardian) is illiterate. A substantial proportion of hospitalized pneumonia patients may not be able to provide informed consent at initial assessment due to confusion or clinical instability. Acutely confused/obtunded patients will be initially recruited on the basis of proxy consent expressed by an accompanying guardian and formal informed consent will be requested when the participant has recovered to the extent that they are able to have reasonable comprehension and retention of the information related to the study.

The main burden for participants is the time and inconvenience of follow up visits at their home. There are also some small risks to the participants from some of the clinical investigations that will be performed (in particular, thoracocentesis, and spirometry) but strict safety guidelines will be followed and will only be performed when no contraindications are present. Study participants will be offered a small gift (up to the value of $4 USD) to compensate them for the time taken and inconvenience of participating in the study.

This study has been approved by the College of Medicine Research Ethics Committee, University of Malawi and the Liverpool School of Tropical Medicine Research Ethics Committee.

## Discussion

Pneumonia is a leading cause of morbidity and mortality in SSA. The AIR Study provides a unique opportunity to study the risk factors for developing pneumonia in a population of individuals in urban Malawi who are carefully characterised during their hospital admission.

The AIR Study is the first study of radiologically confirmed pneumonia in which air pollution exposure measurements have been undertaken in this setting. Although these measurements are limited to a 48 hour snapshot, the measurement of both ambulatory and household CO2 and PM2.5, combined with detailed exposure questionnaires, will contribute important new information about exposure to air pollution in urban SSA.

Through use of already established symptom burden questionnaires for CRD (BOLD questionnaires) and spirometry performed according to ATS/ERS guidelines, the AIR study will provide data that will allow comparison of the burden of CRD across other parts of SSA and further afield, as well as being able to robustly address the primary objective of assessing the contribution of CRD to pneumonia burden. Through identification of preventable risk factors, the AIR Study aims to facilitate future research and implementation of targeted interventions to reduce the high burden of pneumonia in SSA.

## Abbreviations

AIDS: Acquired immunodeficiency syndrome
AIR: Acute infection of the respiratory tract
BinaxNOW® *Streptococcus pneumoniae*: Assay for the rapid detection of *Streptococcus pneumonia* antigen
BMI: Body mass index
BOLD: Burden of obstructive lung disease
CD4: Cluster of differentiation 4
CO: Carbon monoxide
COPD: Chronic obstructive pulmonary disease
CRD: Chronic respiratory disease
DALY: Disability adjusted life year
FEV_1_: Forced expiratory volume in 1 s
FVC: Forced vital capacity
GeneXpert® MTB/RIF: Assay for the rapid detection of tuberculosis and rifampicin resistance
GPS: Global positioning system
HAP: Household air pollution
HIV: Human immunodeficiency virus
PM_2.5_: Particulate matter <2.5 μm
QECH: Queen Elizabeth Central Hospital
SSA: Sub-Saharan Africa
TB: Tuberculosis
UCB-PATS: University of California, Berkley – particle and temperature sensor
WHO: World Health Organisation
